# Supplementary material for: Phylogenetically and catabolically diverse diazotrophs reside in deep-sea cold seep sediments
Source: Nat Commun. 2022 Aug 19;13:4885. doi: 10.1038/s41467-022-32503-w (PMC9391474; doi:10.1038/s41467-022-32503-w)
Supplement: Supplementary file 1 — Supplementary Information [file 41467_2022_32503_MOESM1_ESM.pdf]

Supplementary Information

Phylogenetically and catabolically diverse diazotrophs reside in deep-sea cold seep sediments

Xiyang Dong[1, 2, 3 #] *, Chuwen Zhang[1, 2 #], Yongyi Peng[1, 2], Hong-Xi Zhang[4, 5], Ling-Dong Shi[6], Guangshan Wei[1], Casey R.J. Hubert[7], Yong Wang[4, 5] *, Chris Greening[8]

[1] Key Laboratory of Marine Genetic Resources, Third Institute of Oceanography, Ministry of Natural Resources, Xiamen, 361005, China

[2] School of Marine Sciences, Sun Yat-Sen University, Zhuhai, 519082, China

[3] Southern Marine Science and Engineering Guangdong Laboratory (Zhuhai), Zhuhai, 519000, China

[4] Institute for Marine Engineering, Shenzhen International Graduate School, Tsinghua University, University Town, Shenzhen, 518055, China

[5] Department of Life Science, Institute of Deep-sea Science and Engineering, Chinese Academy of Sciences, Sanya, 572000, China

[6] College of Environmental and Resource Sciences, Zhejiang University, Hangzhou, 310058, China

[7] Department of Biological Sciences, University of Calgary, Calgary, AB T2N 1N4, Canada

[8] Department of Microbiology, Biomedicine Discovery Institute, Clayton, VIC, 3800, Australia

[#] These authors contributed equally to this work.

* Correspondence can be addressed to Xiyang Dong (dongxiyang@tio.org.cn) and Yong Wang (wangyong@sz.tsinghua.edu.cn).

Supplementary Notes

$\delta^{15}N$ records of bulk sediment organic matter from published literature

The nitrogenase reaction induces a large degree of nitrogen isotopic fractionation due to the preferred utilization of ^{14}N, resulting in ^{15}N depletion in sedimentary organic nitrogen[1-3]. On average, lighter $\delta^{15}N$ values were observed in active seep sites than in reference areas lacking signatures of seepage (Supplementary Figure 1). These trends were significant in the sites where the sample size is sufficient for statistical tests to be performed (Site F and Haima; $P < 0.05$ and $P < 0.001$, two-sided Student's t-test, respectively). Overall, lighter sedimentary $\delta^{15}N$ values found at various types of cold seeps suggest nitrogen assimilation through microbial nitrogen fixation[2]. It cannot be ruled out that external reduced nitrogen with low $\delta^{15}N$ signatures, for example from isotopically light ammonium, may have also contributed to these lower values[4-6].

Supplementary Figures

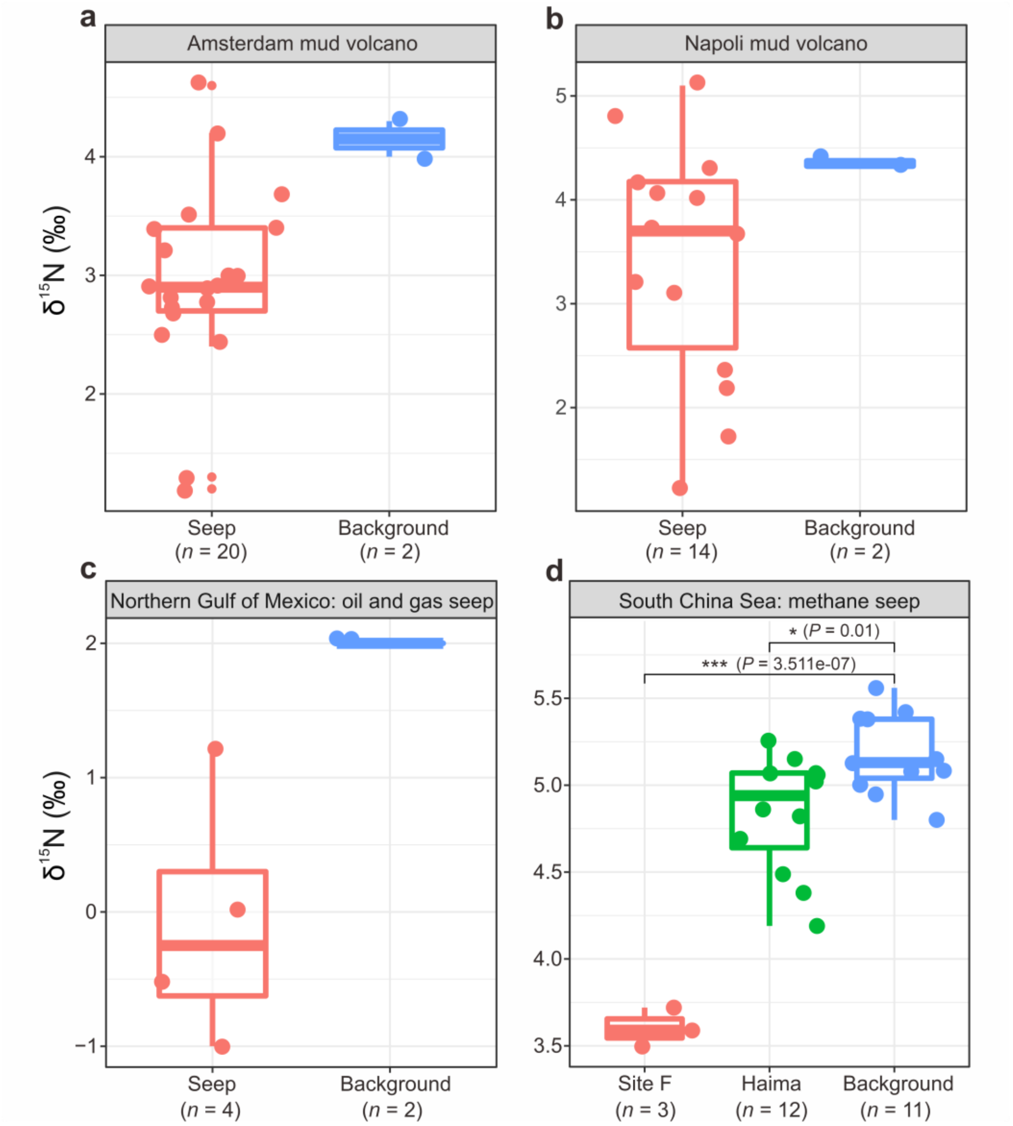

Supplementary Figure 1. A literature summary of $\delta^{15}N$ records of bulk sediment organic matter from five active cold-seep sites. Cold seep sediments are compared to nearby background sites where no seepage signals were detected. Nitrogen stable isotope values are reported in per mil (‰) relative to atmospheric N_2 (air). *n* values refer to the number of biologically independent samples for statistics analysis. Asterisks indicate statistically significant differences between cold seep and background groups (two-sided Student's t-test; * for $P < 0.05$, ** for $P < 0.01$ and *** for $P < 0.001$). Boxplot components: center line, median values; box limits, upper and lower quartiles; whiskers, 1.5× interquartile range; points, outliers. Additional details on $\delta^{15}N$ values, cold seep sites and references are presented in Supplementary Data 11.

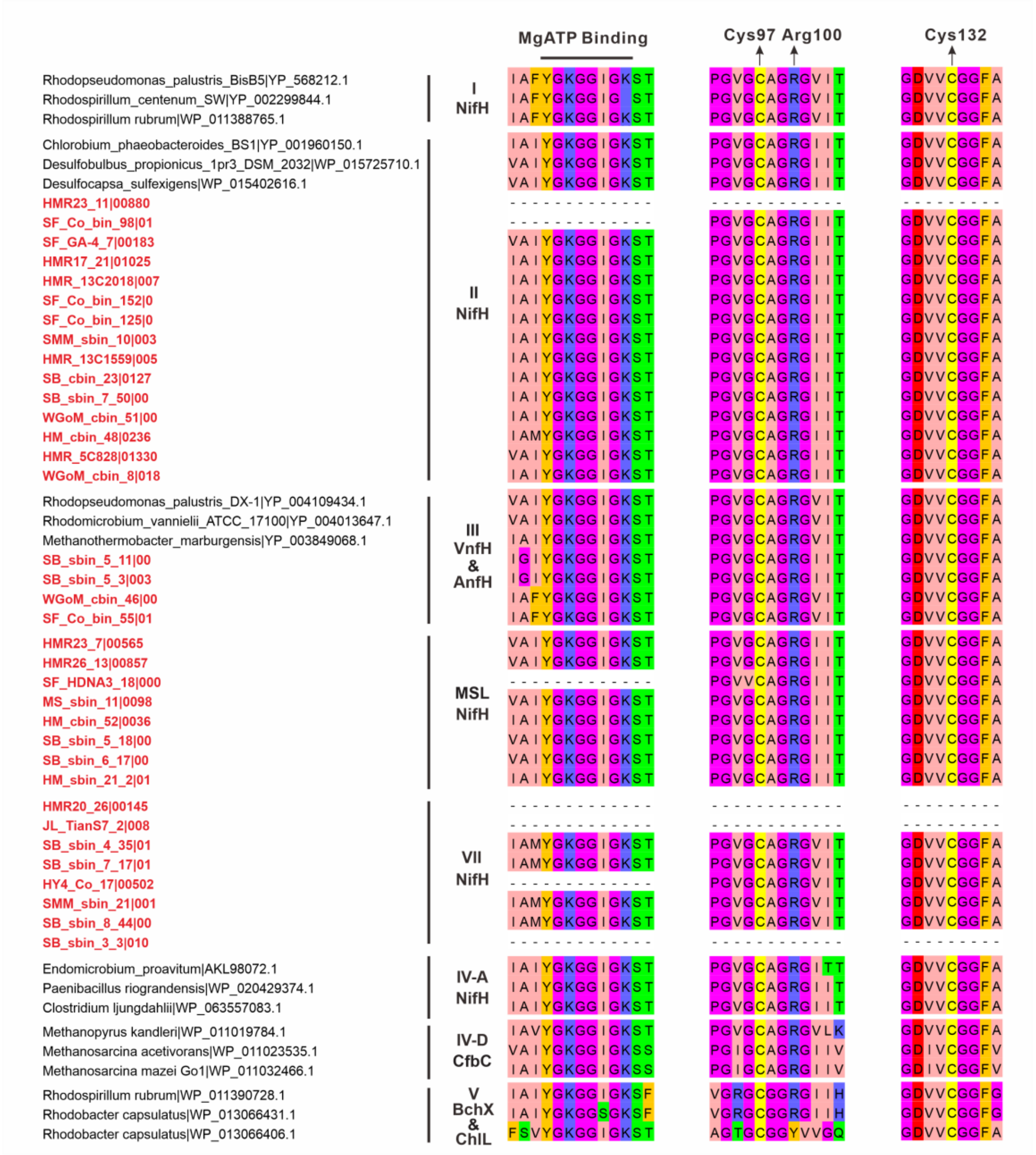

Supplementary Figure 2. NifH superfamily amino acid alignment. Pairwise alignments of NifH superfamily sequences are shown in the region of active site residues responsible for MgATP binding and hydrolysis Fe4-S4 iron sulfur cluster binding. MSL: *Methanosarcina*-like group.

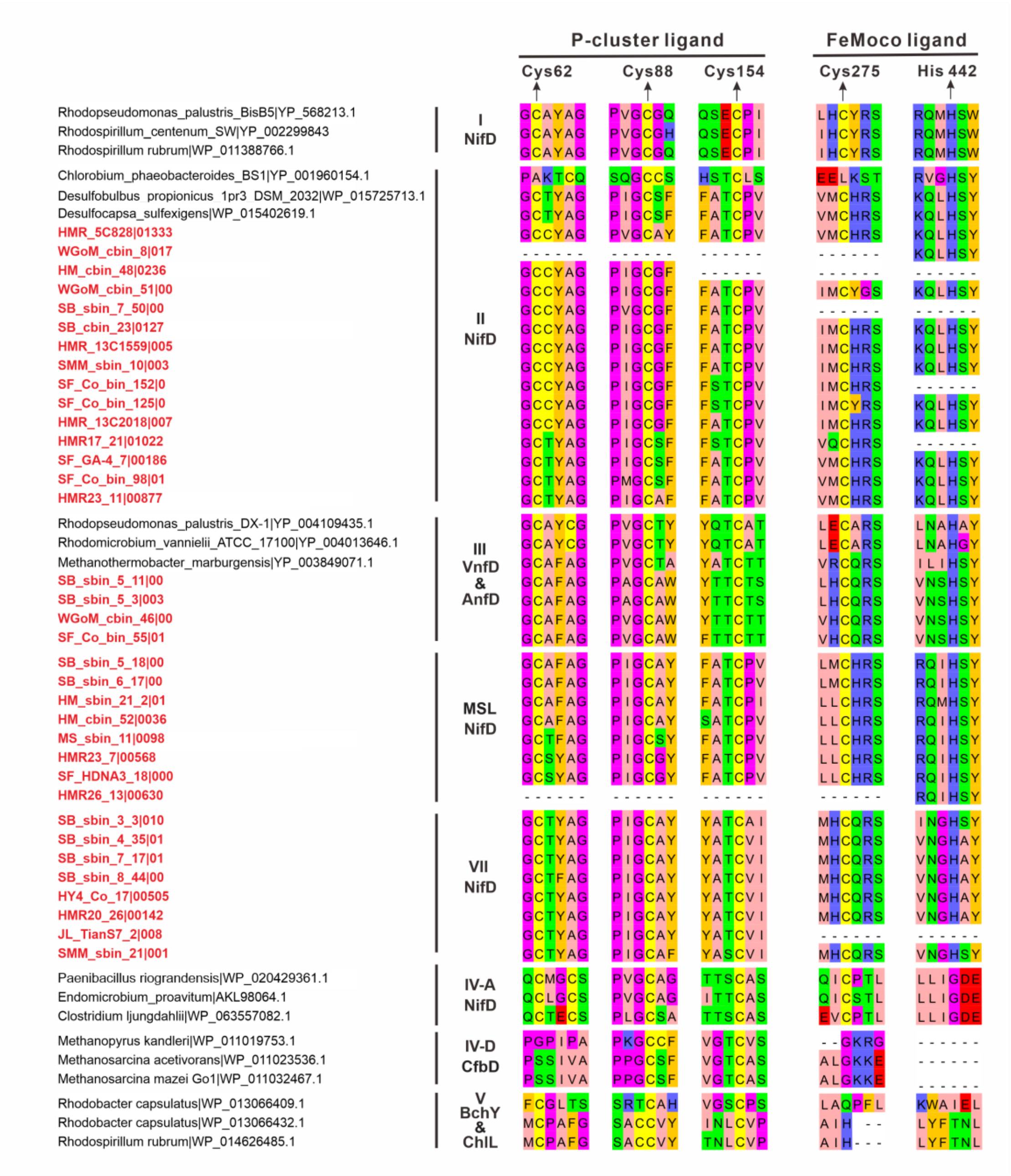

Supplementary Figure 3. NifD superfamily amino acid alignment. Pairwise alignments of NifD superfamily sequences are shown in the region of active site residues responsible for coordination of the P-cluster and FeMo-cofactor within the molybdenum nitrogenase subunit NifD, and substrate bound to the FeMo-cofactor. MSL: *Methanosarcina*-like group.

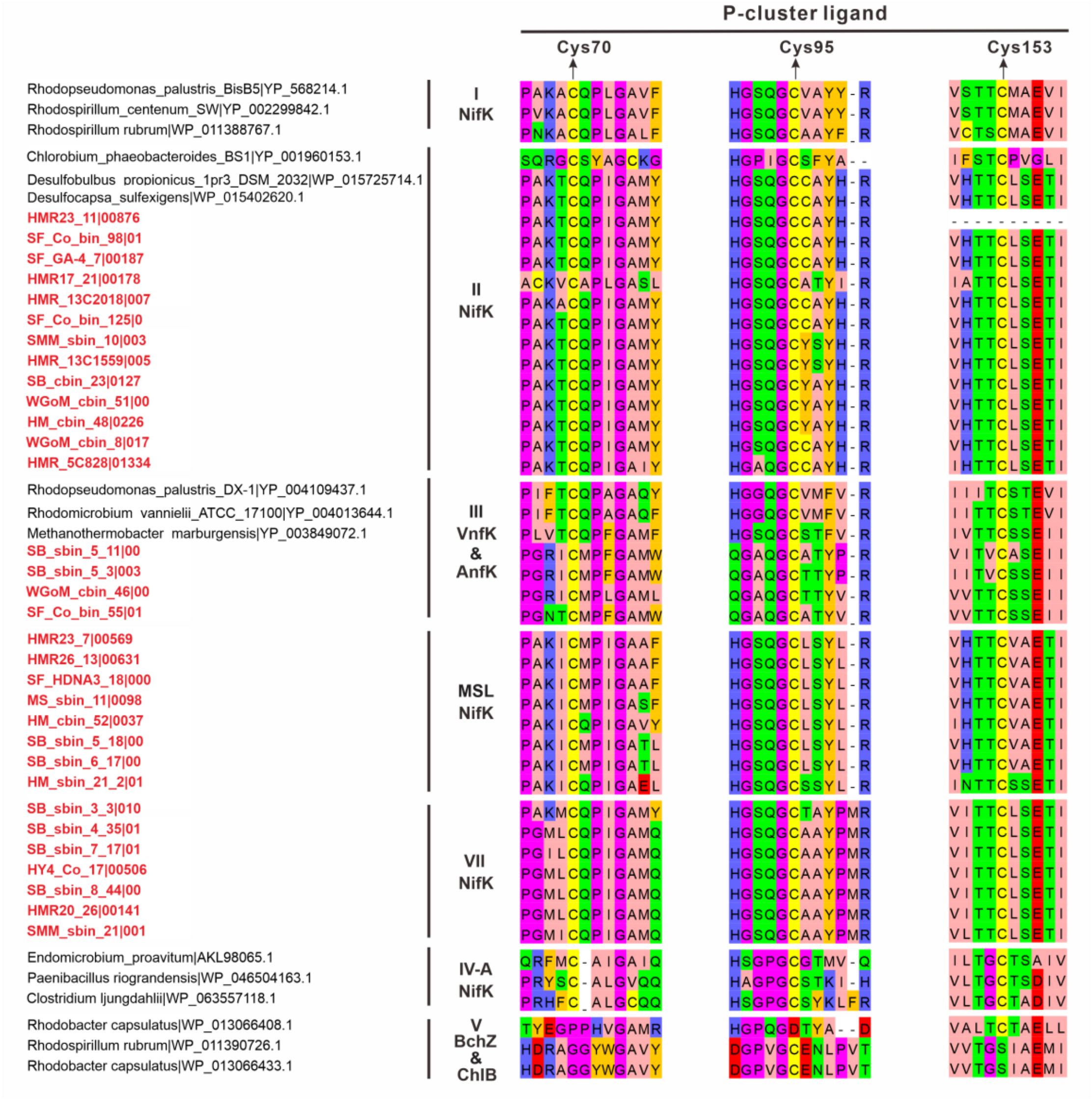

Supplementary Figure 4. NifK superfamily amino acid alignment. Pairwise alignments of NifK superfamily sequences are shown in the region of active site residues responsible for coordination of the P-cluster within the molybdenum nitrogenase subunit NifK. MSL: *Methanosarcina*-like group.

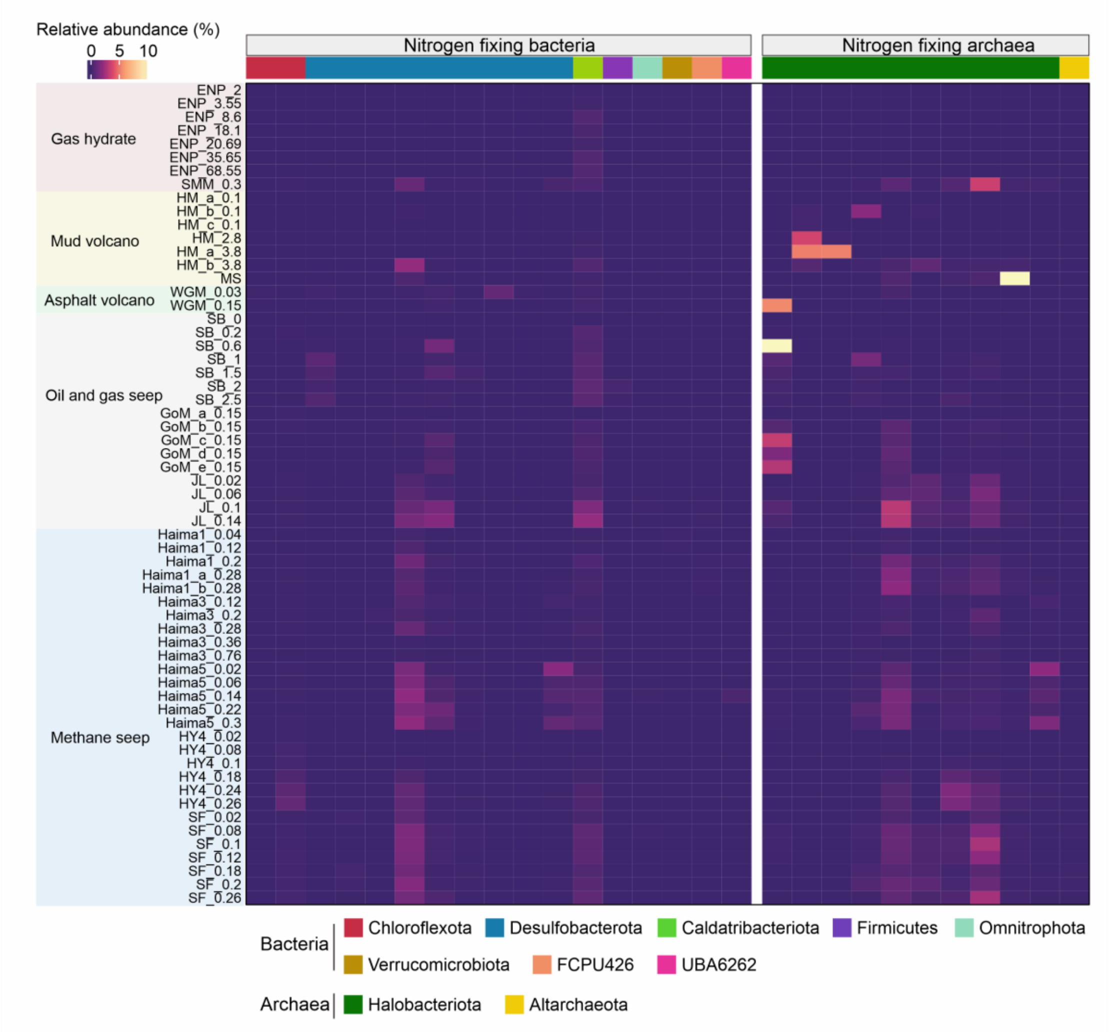

Supplementary Figure 5. Relative abundance of dereplicated nitrogen-fixing MAGs across various cold seep sediment samples. Details on the abundance of nitrogen-fixing MAGs can be found in Supplementary Data 5.

References

1. Zhang, X., Sigman, D.M., Morel, F.M. & Kraepiel, A.M. Nitrogen isotope fractionation by alternative nitrogenases and past ocean anoxia. *Proc Natl Acad Sci U S A* **111**, 4782-4787 (2014).
2. Wada, E. & Hattori, A. Natural abundance of ^{15}N in particulate organic matter in the North Pacific Ocean. *Geochim Cosmochim Acta* **40**, 249-251 (1976).
3. Zerkle, A.L., Junium, C.K., Canfield, D.E. & House, C.H. Production of ^{15}N-depleted biomass during cyanobacterial N_2-fixation at high Fe concentrations. *J Geophys Res* **113** (2008).
4. Philippi, M. et al. Purple sulfur bacteria fix N_2 via molybdenum-nitrogenase in a low molybdenum Proterozoic ocean analogue. *Nat Commun* **12**, 4774 (2021).
5. Vo, J., Inwood, W., Hayes, J.M. & Kustu, S. Mechanism for nitrogen isotope fractionation during ammonium assimilation by *Escherichia coli* K12. *Proc Natl Acad Sci U S A* **110**, 8696-8701 (2013).
6. Hu, Y. et al. A prominent isotopic fingerprint of nitrogen uptake by anaerobic methanotrophic archaea. *Chem Geol* **558**, 119972 (2020).
