# Supplementary material for: Phylogenetically and catabolically diverse diazotrophs reside in deep-sea cold seep sediments
Source: Nat Commun. 2022 Aug 19;13:4885. doi: 10.1038/s41467-022-32503-w (PMC9391474; doi:10.1038/s41467-022-32503-w)
Supplement: Supplementary file 3 — Description of Additional Supplementary Files [file 41467_2022_32503_MOESM3_ESM.docx]

**Description of Additional Supplementary Files**

**File Name:** Supplementary Data 1

**Description:** Sampling and sequencing information for metagenomic and metatranscriptomic data used in this study, including geographic location of cold seeps, cold seep types, sample depths, water depths, sequencing platform, data size, NCBI accession numbers, and source references.

**File Name:** Supplementary Data 2

**Description:** Relative abundances of *nifH* and the oxidative *mcrA* genes used for drawing Figure 3c. The percentages were calculated by dividing the RPKM (Reads per kilo base per million mapped reads) value of *nifH* genes by the mean RPKM value estimated from 14 single-copy marker genes.

**File Name:** Supplementary Data 3

**Description:** Taxonomic assignments of 1428 non-redundant bacterial (*n* = 1146) and archaeal (*n* = 282) population genomes.

**File Name:** Supplementary Data 4

**Description:** Genome statistics of 35 nitrogen-fixing MAGs.

**File Name:** Supplementary Data 5

**Description:** Relative abundance of dereplicated nitrogen-fixing MAGs used for drawing Supplementary Figure 5.

**File Name:** Supplementary Data 6

**Description:** Functional annotation results based on METABOLIC.

**File Name:** Supplementary Data 7

**Description:** Detailed annotations used to draw Figure 5.

**File Name:** Supplementary Data 8

**Description:** Functional annotation results based on DRAM.

**File Name:** Supplementary Data 9

**Description:** Presence of various hydrogenases in the recovered 1428 MAGs.

**File Name:** Supplementary Data 10

**Description:** Expression of *nifH* genes in Haima and Jiaolong cold seep sediments, in the unit of Transcripts Per Million (TPM).

**File Name:** Supplementary Data 11

**Description:** δ^15^N records of bulk sediment organic matter from five active cold-seep sites used for drawing Supplementary Figure 1. Cold seep sites include the Napoli and Amsterdam mud volcanoes in the eastern Mediterranean Sea, an oil and gas seep in the Northern Gulf of Mexico, and methane seeps of Site F and Haima in the South China Sea, with the latter two also near gas hydrate deposits. Geographic location, cold seep types, sediment depth, C/N ratio, and source reference were also included.
